# Hybrid controller with neural network PID/FOPID operations for two-link rigid robot manipulator based on the zebra optimization algorithm

**DOI:** 10.3389/frobt.2024.1386968

**Published:** 2024-06-14

**Authors:** Mohamed Jasim Mohamed, Bashra Kadhim Oleiwi, Ahmad Taher Azar, Ahmed Redha Mahlous

**Affiliations:** ^1^ Control and Systems Engineering Department, University of Technology-Iraq, Baghdad, Iraq; ^2^ College of Computer and Information Sciences, Prince Sultan University, Riyadh, Saudi Arabia; ^3^ Automated Systems and Soft Computing Lab (ASSCL), Prince Sultan University, Riyadh, Saudi Arabia; ^4^ Faculty of Computers and Artificial Intelligence, Benha University, Benha, Egypt

**Keywords:** neural network, recurrent neural network, set-point controller, proportional integral derivative controller, fractional-order PID controller, 2-link rigid robot manipulator, zebra optimization algorithm

## Abstract

The performance of the robotic manipulator is negatively impacted by outside disturbances and uncertain parameters. The system’s variables are also highly coupled, complex, and nonlinear, indicating that it is a multi-input, multi-output system. Therefore, it is necessary to develop a controller that can control the variables in the system in order to handle these complications. This work proposes six control structures based on neural networks (NNs) with proportional integral derivative (PID) and fractional-order PID (FOPID) controllers to operate a 2-link rigid robot manipulator (2-LRRM) for trajectory tracking. These are named as set-point-weighted PID (W-PID), set-point weighted FOPID (W-FOPID), recurrent neural network (RNN)-like PID (RNNPID), RNN-like FOPID (RNN-FOPID), NN+PID, and NN+FOPID controllers. The zebra optimization algorithm (ZOA) was used to adjust the parameters of the proposed controllers while reducing the integral-time-square error (ITSE). A new objective function was proposed for tuning to generate controllers with minimal chattering in the control signal. After implementing the proposed controller designs, a comparative robustness study was conducted among these controllers by altering the initial conditions, disturbances, and model uncertainties. The simulation results demonstrate that the NN+FOPID controller has the best trajectory tracking performance with the minimum ITSE and best robustness against changes in the initial states, external disturbances, and parameter uncertainties compared to the other controllers.

## 1 Introduction

The field of robotics mainly focuses on problems related to visualization, modeling, and control. Robots are used in many daily tasks and occupations in every aspect of modern life. Robotic manipulators are increasingly required in factories and industries as they play important roles in the operations instead of humans, especially when these operations involve risky, repetitive, and complex activities (Oleiwi et al., 2021). The use of robots has also become necessary to ensure efficient, quick, and accurate operations. Traditional robots are large and bulky since they contain stiff linkages throughout their construction; most industries need upgrades to the current classical robots to lower the building costs, minimize energy consumption brought on by the large actuators, and boost production (Alandoli and Tian, 2020). Since robotic manipulators are well-suited for many applications, particularly in the industrial field, they have been widely used for many years. The trajectory tracking control is an important issue from the viewpoint of automatic control because various applications, such as welding, screwing, moving cars or equipment parts, and painting, demand precise trajectory tracking to accomplish their objectives (Azar and Fernando, 2019). The complexity and non-linearity of a robotic manipulator make it impossible for proportional integral derivative (PID) controllers to provide effective trajectory tracking and constant force/twist control simultaneously. The robotic manipulator also experiences a number of uncertainties, external disturbances, payload variations, and parameter variations during operation (Dachang et al., 2020; Abdulameer and Mohamed, 2022). To design controllers that can handle the dynamics of the manipulator robot for controlling and trajectory tracking, many solutions have been proposed using traditional control systems (Ajeil et al., 2020; Ibraheem et al., 2020; Najm et al., 2020). Sharma et al. (2015) described the design and analysis of a fractional-order PID (FOPID) controller with two degrees of freedom (DOFs) based on the cuckoo search algorithm for a two-link rigid robot manipulator (2-LRRM) with a payload; their results indicated that the suggested strategy improves the performance of the closed-loop system by resolving robustness and disturbance rejection issues. Kumar (2017) proposed an interval type-2 fuzzy proportional derivative plus integral controller based on the genetic algorithm for a 5-DOF redundant robot manipulator. Cao et al. (2021) introduced an invariant control structure for manipulator robot trajectory tracking with input saturation and uncertainty by combining reinforcement learning and non-singular terminal sliding-mode control. Shuyang and John (2021) applied a neural network (NN) as a multilayer perceptron structure based on the iterative process of learning; here, the desired robot joint was employed as the input, and the desired robot motion was related to the output; the movement of the robot with respect to the intended set of joint paths is determined by the iterative learning control. Shojaei et al. (2020) suggested a specific performance-based adaptive neural control system for manipulator robots without considering the input current, acceleration, or velocity; this scheme includes the actuator dynamics under model uncertainty, and an acceleration velocity observer was coupled with a neural adaptive second-order PID controller. Nohooji (2020) addressed the unknown dynamics and outside disturbances of a manipulator robot to design a control approach utilizing NN-based radial basis activation functions and self-tuning PID control. Zhou et al. (2020) and Kareem et al. (2023) introduced fractional-order sliding-mode control using a deep convolutional NN for controlling trajectory tracking in manipulator robots; here, the controller switching gain decreased drastically since the NN corrects the uncertainty of the system without knowledge of the upper boundaries. Four distinguished non-linear control structures were studied by Jenhani et al. (2022) to address the problem of controlling and stabilizing robotic systems to predefined positions. To control the position and velocity of the 2-link robot, classical and adaptive sliding-mode controllers were introduced by Al-Hadithy and Hammoudi (2020) as well as Hameed and Hamoudi (2023). Hamoudi and Rasheed (2023) used particle swarm optimization to study the effectiveness of adaptive and classical backstepping control schemes for non-linear systems.

It is well-known in intelligent control that the NN controller can be used to solve various control issues, particularly when the controlled plant displays non-linearity and/or uncertainties in the model parameters. The advantage of the NN is that it has solid capability for mapping. Conversely, the PID controller is the most widely used controller in the industry because of its robust performance under numerous operating conditions and straightforward design. Therefore, in our proposed controllers, we merge the advantages of NNs with those of the PID and FOPID controllers to obtain hybrid controllers based on the zebra optimization algorithm (ZOA) to control a 2-LRRM.

The following are the main contributions of this work:

1. Six control structures are proposed based on NNs with PID/FOPID operations.
2. ZOA is used to adjust the gains of the proposed control structures on the basis of reducing the integral-time-square error (ITSE) performance index.
3. A comparative robustness study is conducted among the proposed control structures by altering the initial conditions, disturbances, and model uncertainties.
4. A new objective function is proposed for the tuning process to obtain controllers with minimal chattering in the control signals.

The remainder of this paper is structured as follows: the 2-LRRM’s mathematical model is described in Section 2. The proposed controller structures are discussed in Section 3. The ZOA is explained in Section 4. The simulation results are shown in Section 5; the robustness tests are presented in Section 6, and the conclusions are presented in Section 7.

## 2 Mathematical model of the 2-LRRM

**FIGURE 1:**
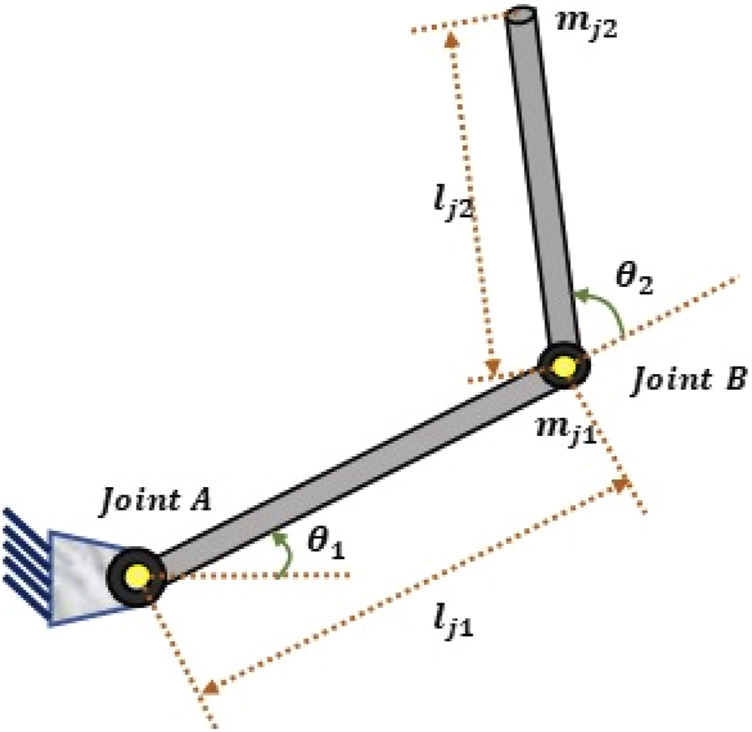
Structure of the 2-LRRM.

The 2-LRRM structure is illustrated in Figure 1. It is composed of two links with lengths $l_{j1}$ and $l_{j2}$ and two mass centers $m_{j1}$ and $m_{j2}$ that are at the distal ends of the links. Encoders determine the angular positions of the links ( $\theta_{1}$ and $\theta_{2}$ ) and velocities ( ${\dot{\theta }}_{1}$ and ${\dot{\theta }}_{2}$ **)**, while the regulating torque is produced at points A and B by DC motors (Mohan et al., 2018). In robotics, the fundamental control equations are constructed using the dynamic equation of robot motion. In a robotic system, the actuator torque is employed to produce the dynamic motion of the manipulator arm. The dynamic modeling of a robotic system is characterized by the connections between the temporal rates of change and input torques of the robot arm component configurations. The primary goal here is to determine how to calculate the robot’s motion equations given the moments and forces exerted on it. Therefore, a part of the robot manipulator’s dynamic modeling describes the joint locations, velocities, and accelerations in addition to the functions mapping the forces acting on the structures (Raafat and Raheem, 2017).

The equations describing the x and y positions of ${\;m}_{j1}$ and ${\;m}_{j2}$ , the equation for the kinetic and potential energies, and the two derived coupled non-linear differential equations based on the Euler–Lagrange equation are presented in Lewis et al. (2004) and Raafat and Raheem (2017).

The conventional form can be applied to the manipulator dynamics as indicated in Eqs. (1 -10)

$$M\;\left(\theta \right)\ddot{\theta }+V\left(\theta ,\dot{\theta }\right)+g\left(\theta \right)=T,$$ (1)

where $V\left(\theta ,\dot{\theta }\right)$ is the Coriolis/centripetal vector, $M\left(\theta \right)$ is the inertia matrix that exhibits symmetry, and $g\left(\theta \right)$ is the gravity vector.

$$M=\left[\begin{array}{cc} M11 & M12\\ M21 & M22 \end{array}\right],$$ (2)

$$M_{11}=\left(m_{j1}+m_{j2}\right)l_{j1}^{2}+m_{j2}l_{j2}^{2}+2m_{j2}l_{j1}l_{j2\;}\cos\left(\theta_{2}\right),$$ (3)

$$M_{12}=m_{j2}l_{j2}^{2}+m_{j2}l_{j1}l_{j2\;}\cos\left(\theta_{2}\right),$$ (4)

$$M_{12}=M_{21}\;\&\;M_{22}=m_{j2}l_{j2}^{2}.$$ (5)

The Coriolis/centrifugal vector denoted by ***V*** is of the form

$$V=\left[\begin{array}{c} V_{1}\\ V_{2} \end{array}\right],$$ (6)

$$V_{1}=-m_{j2}l_{j1},$$ (7)

$$V_{2}=m_{j2}l_{j1}l_{j2}{\dot{\theta }}_{1}^{2}\sin\left(\theta_{2}\right).$$ (8)

The gravity vector $g={\left[{\;g}_{12\;}g_{21\;}\right]}^{T}$ is defined using

$$g_{12}=\left(m_{j1}+m_{j2}\right)gl_{j1}\cos\left(\theta_{1}\right)+m_{j2}gl_{j2}\cos\;\left(\theta_{1}+\theta_{2}\right),$$ (9)

$$g_{21}=m_{j2}gl_{j2}\cos\left(\theta_{1}+\theta_{2}\right).$$ (10)

The 2-LRRM parameter specifications are as listed in Table 1 (Mohamed et al., 2023).

**TABLE 1:** Specifications of the 2-LRRM.

Parameter	Nominal value
$m_{j1}$	0.1 kg
$m_{j2}$	0.1 kg
$l_{j1}$	0.8 m
$l_{j2}$	0.4 m
g	9.81 m/ $s^{2}$

## 3 Structures of the proposed hybrid controllers

This section provides the descriptions of the proposed controllers below.

### 3.1 Set-point-weighted PID and FOPID controllers

**FIGURE 2:**
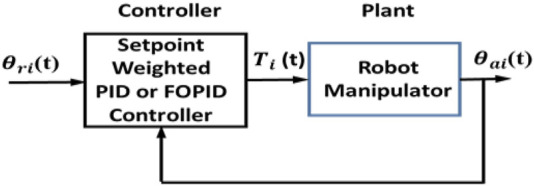
Set-point-weighted controller block diagram.

This section discusses set-point-weighted PID and FOPID controllers. The block diagram of this feedback control system is indicated in Figure 2.

The equation describing the set-point-weighted PID (W-PID) controller is presented in Eq. (11):

$$\begin{array}{ll} {T_{i}\left(t\right)}_{PID} & =K_{p}\left(\left(1-\beta \right)\theta_{ri}\left(t\right)-\theta_{ai}\left(t\right)\right)+K_{i}\int \left(\theta_{ri}\left(t\right)-\theta_{ai}\left(t\right)\right)\;dt\\ & \;+K_{d}\frac{d}{dt}\left(\left(1-\alpha \right)\theta_{ri}\left(t\right)-\theta_{ai}\left(t\right)\right), \end{array}$$ (11)

where ${\;e_{\theta i}\left(t\right)=\theta }_{ri}\left(t\right)-\theta_{ai}\left(t\right),0<\beta <1$ ***,*** 0<α<1 .

Here, $e_{\theta i}\left(t\right)$ is the error between the required and calculated positions, $\theta_{ri}\left(t\right)$ and $\theta_{ai}\left(t\right)$ , respectively, of the i th link; $K_{p},K_{i}$ , and $K_{d}$ are the proportional, integral, and derivative gains of the PID controller, respectively; $T_{i}\left(t\right)$ is the control signal (torque) of the i th link; β and α are constants. The equation of the set-point-weighted FOPID (W-FOPID) controller contains two additional parameters ( λ ***,*** μ ) with fractional values, as shown in Eq. (12):

$$\begin{array}{ll} {T_{i}\left(t\right)}_{FOPID} & =K_{p}\left(\left(1-\beta \right)\theta_{ri}\left(t\right)-\theta_{ai}\left(t\right)\right)+K_{i}D^{-\lambda }\left(\theta_{ri}\left(t\right)-\theta_{ai}\left(t\right)\right)\\ & \;+K_{d}D^{\mu }\left(\left(1-\alpha \right)\theta_{ri}\left(t\right)-\theta_{ai}\left(t\right)\right), \end{array}$$ (12)

where 0<λ<2 ***,*** 0<μ<2 ***,*** and ***D*** represents the Laplace variable.

In Eqs. (11, 12), α and β are used to adjust the set-point values before comparing with the output to calculate the error signal for each control action. Here, β is used to attenuate the set-point signal before computing the corresponding error for the proportional action and α is used similarly for the derivative action, while the set-point signal attenuation is not used for the integral action. This PID configuration is unconventional, famous, and used in many works. 

**FIGURE 3:**
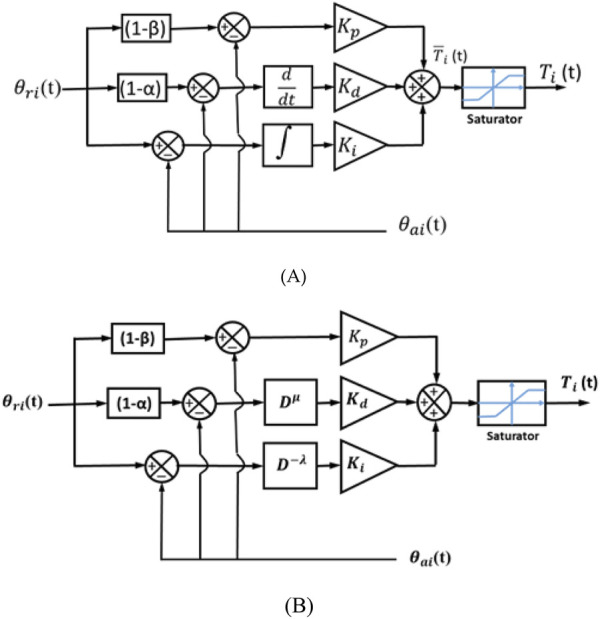
Set-point-weighted controller structures: **(A)** W-PID and **(B)** W-FOPID.

The set-point-weighted PID and FOPID controller structures are demonstrated in Figure 3.

### 3.2 Recurrent neural network (RNN)-like PID and FOPID controllers

**FIGURE 4:**
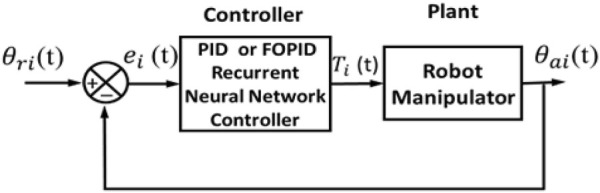
Recurrent-neural-network-like PID and FOPID controller block diagrams (RNN-PID and RNN-FOPID).

**FIGURE 5:**
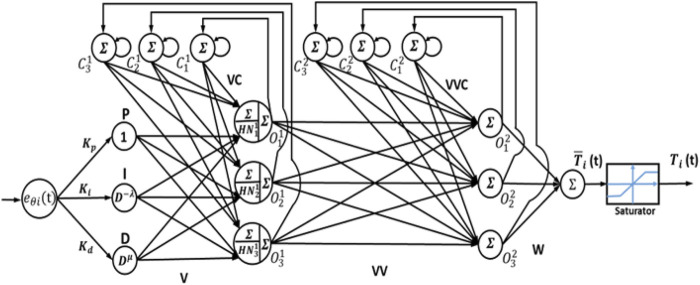
Recurrent-neural-network-like PID and FOPID structures (RNN-PID and RNN-FOPID controllers).

In these two controllers, RNN-like PID and FOPID are adopted. The block diagram of the feedback control system for this type of hybrid controller is shown in Figure 4. The common structure of the RNN-PID and RNN-FOPID controllers is shown in Figure 5. For the conventional PID controller, the order variables have integer values λ=1 and μ=1 **.** ${\;e}_{\theta i}\left(t\right)$ is the error between the required and actual positions $\theta_{ri}\left(t\right)$ and $\theta_{ai}\left(t\right)$ , respectively, of the ith link.

In the RNN-PID controller, the input layer has a single neuron $e_{\theta i}\left(t\right)$ . The first hidden layer has three neurons ***P***, ***I***, and ***D*** that are defined in Eqs (13–15):

$$P\left(t\right)=K_{p}e_{\theta i}\left(t\right)\;or\;P\left(k\right)=K_{p}e_{\theta i}\left(k\right),$$ (13)

$$I\left(t\right)=K_{i}\int e_{\theta i}\left(t\right)\;dt\;or\;I\left(k\right)=K_{i}\sum_{j=0}^{k}{\;e}_{i}\left(j\right),$$ (14)

$$D\left(t\right)=K_{d}\;\frac{d}{dt}e_{\theta i}\left(t\right)\;or\;D\left(k\right)=K_{d}\;\left(e_{\theta i}\left(k\right)-e_{\theta i}\left(k-1\right)\;\right)/h,$$ (15)

where, the feedback and processing elements in the second hidden layer are defined in Eqs. (16 -17)

$$\left[\begin{array}{c} N_{1}^{1}\left(k\right)\\ N_{2}^{1}\left(k\right)\\ N_{3}^{1}\left(k\right)\; \end{array}\right]=\left[\begin{array}{ccc} v11 & v12 & v13\\ \;v21\; & v22 & \;v23\;\\ v31 & v32 & v33 \end{array}\right]\left[\begin{array}{c} P\left(k\right)\\ I\left(k\right)\\ D\left(k\right) \end{array}\right]$$ (16)

and

$$\left[\begin{array}{c} C_{1}^{1}\left(k\right)\\ C_{2}^{1}\left(k\right)\\ C_{3}^{1}\left(k\right)\; \end{array}\right]=\left[\begin{array}{c} O_{1}^{1}\left(k-1\right){+p1\;C}_{1}^{1}\left(k-1\right)\\ O_{2}^{1}\left(k-1\right){+p2\;C}_{2}^{1}\left(k-1\right)\\ {\;O}_{3}^{1}\left(k-1\right)\;{+p3\;C}_{3}^{1}\left(k-1\right)\; \end{array}\right].$$ (17)

The output of the second hidden layer is given by Eq. (18):

$$\left[\begin{array}{c} O_{1}^{1}(k\\ O_{2}^{1}\left(k\right)\\ {\;O}_{3}^{1}\left(k\right)\; \end{array}\right]=\left[\begin{array}{c} \left.H{\left(N\right.}_{1}^{1}\left(k\right)\right)\\ \left.H{\left(N\right.}_{2}^{1}\left(k\right)\right)\\ H\left(N_{3}^{1}\left(k\right)\right) \end{array}\right]+\left[\begin{array}{ccc} vc11 & vc12 & vc13\\ \;vc21\; & vc22 & \;vc23\;\\ vc31 & vc32 & vc33 \end{array}\right]\left[\begin{array}{c} C_{1}^{1}\left(k\right)\\ C_{2}^{1}\left(k\right)\\ C_{3}^{1}\left(k\right)\; \end{array}\right].$$ (18)

The activation function used is a sigmoid function, as shown in Eq. (19):

$$H=\frac{2}{\left(1+e^{-net}\right)}-1,$$ (19)

where, the feedback elements in the third hidden layer are defined in Eq. (20)

$$\left[\begin{array}{c} C_{1}^{2}\left(k\right)\\ C_{2}^{2}\left(k\right)\\ C_{3}^{2}\left(k\right)\; \end{array}\right]=\left[\begin{array}{c} O_{1}^{2}\left(k-1\right){+pp1\;C}_{1}^{2}\left(k-1\right)\\ O_{2}^{2}\left(k-1\right){+pp2\;C}_{2}^{2}\left(k-1\right)\\ {\;O}_{3}^{2}\left(k-1\right){+pp3\;C}_{3}^{2}\left(k-1\right)\; \end{array}\right].$$ (20)

The output of the third hidden layer is expressed in Eq. (21):

$$\left[\begin{array}{c} O_{1}^{2}\left(k\right)\\ O_{2}^{2}\left(k\right)\\ {\;O}_{3}^{2}\left(k\right) \end{array}\right]=\left[\begin{array}{ccc} vv11 & vv12 & vv13\\ \;vv21\; & vv22 & \;vv23\;\\ vv31 & vv32 & vv33 \end{array}\right]\left[\begin{array}{c} O_{1}^{1}(k\\ O_{2}^{1}\left(k\right)\\ {\;O}_{3}^{1}\left(k\right) \end{array}\right]+$$

$$\left[\begin{array}{ccc} vvc11 & vvc12 & vvc13\\ \;vvc21\; & vvc22 & \;vvc23\;\\ vvc31 & vvc32 & vvc33 \end{array}\right]\left[\begin{array}{c} C_{1}^{2}\left(k\right)\\ C_{2}^{2}\left(k\right)\\ C_{3}^{2}\left(k\right)\; \end{array}\right].$$ (21)

The output of the single neuron in the output layer is given in Eq. (22):

$$T_{i}\left(k\right)=w_{1}\;O_{1}^{2}\left(k\right)+w_{2}\;O_{2}^{2}\left(k\right)+w_{3}\;O_{3}^{2}\left(k\right),$$ (22)

where $K_{p},K_{i},K_{d},vij,vcij\;vvij,vvcij,w_{i},p_{i},\text{and }pp_{i}$ are all parameters.

In the RNN-FOPID controller, the input layer again has a single neuron ${\;e}_{\theta i}\left(t\right)$ **.** The first hidden layer has three neurons ***P, I,*** and ***D***, where the order variables 0<λ<2 and 0<μ<2 are fractions instead of integers, and the ***P, I,*** and ***D*** neurons of this hidden layer are shown in Eqs (23–25). All the remaining hidden layers and output layer are the same as in the RNN-PID controller.

$$P\left(t\right)=K_{p}\;e_{\theta i}\left(t\right),$$ (23)

$$I\left(t\right)=K_{i\;}D^{-\lambda }\;e_{\theta i}\left(t\right),$$ (24)

$$D\left(t\right)=K_{d}\;D^{\mu }\;e_{\theta i}\left(t\right).$$ (25)

### 3.3 NN-based PID and FOPID controllers

**FIGURE 6:**
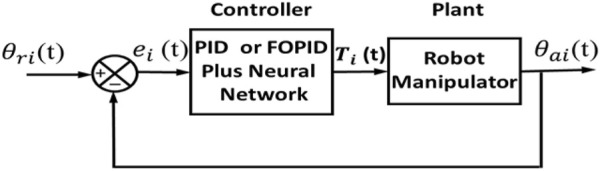
Block diagram of a neural network combined with PID and FOPID controllers.

In this type of controller, the NN and PID/FOPID controllers both contribute to the production of the control signal. Figure 6 displays the feedback control system block diagram for this type of controller. 

**FIGURE 7:**
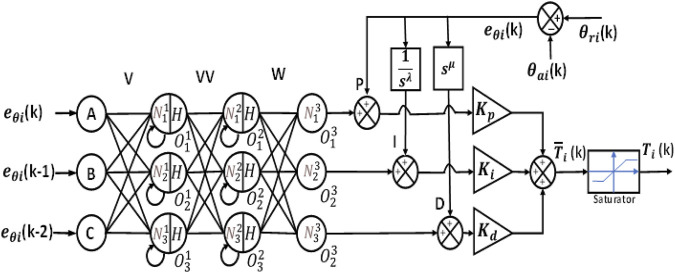
Neural network combined with PID and FOPID controller structures (NN + PID and NN + FOPID).

The common structure for these two controllers is indicated in Figure 7.

For the conventional PID controller, the order variables have integer values λ=1 and μ=1 . There are three neurons in the input layer of the NN + PID controller structure, namely, $e_{\theta i}\left(k\right),e_{\theta i}\left(k-1\right),\text{and }e_{\theta i}\left(k-2\right)$ or A, B, and C.

Thus, the first layer of hidden neurons is given by Eq. (26):

$$\left[\begin{array}{c} N_{1}^{1}\left(k\right)\\ N_{2}^{1}\left(k\right)\\ N_{3}^{1}\left(k\right)\; \end{array}\right]=\left[\begin{array}{ccc} v11 & v12 & v13\\ \;v21\; & v22 & \;v23\;\\ v31 & v32 & v33 \end{array}\right]\left[\begin{array}{c} e_{\theta i}\left(k\right)\\ e_{\theta i}\left(k-1\right)\\ e_{\theta i}\left(k-2\right) \end{array}\right]+\left[\begin{array}{c} N_{1}^{1}\left(k-1\right)\\ N_{2}^{1}\left(k-1\right)\\ N_{3}^{1}\left(k-1\right)\; \end{array}\right].$$ (26)

The output of the first hidden layer is given by Eq. (27):

$$\left[\begin{array}{c} O_{1}^{1}\left(k\right)\\ O_{2}^{1}\left(k\right)\\ O_{3}^{1}\left(k\right)\; \end{array}\right]=\left[\begin{array}{c} H\left(N_{1}^{1}\left(k\right)\right)\\ \left.{H(N}_{2}^{1}\left(k\right)\right)\\ {H(N}_{3}^{1}\left(k\right))\; \end{array}\right],$$ (27)

$$\left[\begin{array}{c} N_{1}^{2}\left(k\right)\\ N_{2}^{2}\left(k\right)\\ N_{3}^{2}\left(k\right)\; \end{array}\right]=\left[\begin{array}{ccc} vv11 & vv12 & vv13\\ \;vv21\; & vv22 & \;vv23\;\\ vv31 & vv32 & vv33 \end{array}\right]\left[\begin{array}{c} O_{1}^{1}\left(k\right)\\ O_{2}^{1}\left(k\right)\\ O_{3}^{1}\left(k\right)\; \end{array}\right]+\left[\begin{array}{c} N_{1}^{2}\left(k-1\right)\\ N_{2}^{2}\left(k-1\right)\\ N_{3}^{2}\left(k-1\right)\; \end{array}\right].$$ (28)

The input and output of the second hidden layer are given by Eqs (28, 29), respectively.

$$\left[\begin{array}{c} O_{1}^{2}\left(k\right)\\ O_{2}^{2}\left(k\right)\\ O_{3}^{2}\left(k\right)\; \end{array}\right]=\left[\begin{array}{c} \left.{H(N}_{1}^{2}\left(k\right)\right)\\ H\left(N_{2}^{2}\left(k\right)\right)\\ H\left(N_{3}^{2}\left(k\right)\right)\; \end{array}\right].$$ (29)

The activation function used is a sigmoid function, as shown in Eq. (30):

$$H=\frac{2}{\left(1+e^{-net}\right)}-1.$$ (30)

The output of the third hidden layer is given by Eq. (31):

$$\left[\begin{array}{c} O_{1}^{3}\left(k\right)\\ O_{2}^{3}\left(k\right)\\ O_{3}^{3}\left(k\right)\; \end{array}\right]=\left[\begin{array}{c} N_{1}^{3}\left(k\right)\\ N_{2}^{3}\left(k\right)\\ N_{3}^{3}\left(k\right)\; \end{array}\right]=\left[\begin{array}{ccc} w11 & w12 & w13\\ w21\; & w22 & \;w23\;\\ w31 & w32 & w33 \end{array}\right]\left[\begin{array}{c} O_{1}^{2}\left(k\right)\\ O_{2}^{2}\left(k\right)\\ O_{3}^{2}\left(k\right)\; \end{array}\right].$$ (31)

Equations (32–34) define the three control actions of the PID controller, and each of these control actions is added to one of the neuron outputs of the NN, as shown in Eqs. (35–37).

$$P\left(t\right)=e_{\theta i}\left(t\right)\;or\;P\left(k\right)={\;e}_{\theta i}\left(k\right),$$ (32)

$$I\left(t\right)=\int e_{\theta i}\left(t\right)\;dt\;or\;I\left(k\right)=\sum_{j=0}^{k}e_{\theta i}\left(j\right),$$ (33)

$$D\left(t\right)=\frac{d}{dt}e_{\theta i}\left(t\right)\;or\;D\left(k\right)=\left(e_{\theta i}\left(k\right)-e_{\theta i}\left(k-1\right)\;\right)/h,$$ (34)

$${u_{1}\left(k\right)=K}_{p}\left(O_{1}^{3}\left(k\right)+{\;e}_{\theta i}\left(k\right)\right),$$ (35)

$${u_{2}\left(k\right)=K}_{i}\left(O_{2}^{3}\left(k\right)+\sum_{j=0}^{k}e_{\theta i}\left(j\right)\right),$$ (36)

$${u_{3}\left(k\right)=K}_{d}\left(O_{3}^{3}\left(k\right)+\left(e_{\theta i}\left(k\right)-e_{\theta i}\left(k-1\right)\right)/h\right).$$ (37)

The equation of the control signal is expressed by Eq. (38):

$${\bar{T}}_{i}\left(k\right)=u_{1}\;\left(k\right)+u_{2}\;\left(k\right)+u_{3}\;\left(k\right).$$ (38)

The structure of the NN + FOPID controller is the same as that of the NN + PID controller, with the difference being fractional-order operations of the integral and derivative functions given by 0<λ<2 and 0<μ<2 , respectively, as illustrated in Eqs. (39–44).

$$P\left(k\right)=e_{\theta i}\left(k\right),$$ (39)

$$I\left(k\right)=D^{-\lambda }\;e_{\theta i}\left(k\right),$$ (40)

$$D\left(k\right)=D^{\mu }\;e_{\theta i}\left(k\right),$$ (41)

$${u_{1}\left(k\right)=K}_{p}\left(O_{1}^{3}\left(k\right)+{\;e}_{\theta i}\left(k\right)\right),$$ (42)

$${u_{2}\left(k\right)=K}_{i}\;\left(O_{2}^{3}\left(k\right)+D^{-\lambda }\;e_{\theta i}\left(k\right)\right),$$ (43)

$${u_{3}\left(k\right)=K}_{d}\left({\;O}_{3}^{3}\left(k\right)+D^{\mu }\;e_{\theta i}\left(k\right)\right).$$ (44)

The equation of the control signal is given by Eq. (45):

$${\bar{T}}_{i}\left(k\right)=u_{1}\;\left(k\right)+u_{2}\;\left(k\right)+u_{3}\;\left(k\right).$$ (45)

## 4 Zebra optimization algorithm

The ZOA is a nature-inspired metaheuristic algorithm and is presented mathematically in this section (Trojovská et al., 2022). The actions of zebras in the wild serve as the primary source of inspiration for the ZOA, where the foraging behaviors and defense mechanisms against predator attacks are simulated. The description is provided first, followed by the mathematical modeling of the ZOA steps. Effective real-world optimization issues can be resolved by the ZOA by achieving an appropriate balance between exploration and exploitation.

• Initialization

The population of zebras that provide a solution to the problem can be numerically modeled using a matrix, in addition to the plain where the zebras are located inside the search space. Within the search area, the zebras are positioned randomly at their starting positions. Equation (46) provides the structure of the ZOA population matrix.

$$X={\left[\begin{array}{c} X_{1}\\ X_{i}\\ X_{N} \end{array}\right]}_{N\times m}={\left[\begin{array}{ccc} x_{1,1} & x_{1,j} & x_{1,m}\\ x_{i,1} & x_{i,j} & x_{i,m}\\ x_{N,1} & x_{N,j} & x_{N,m} \end{array}\right]}_{N\times m},$$ (46)

where ***X*** is the population, *N* is the total number of initial solutions in the population, and *m* is the number of variables in each solution; $X_{i}$ is the ith solution, and $x\left(i,j\right)$ is the value of the jth problem variable proposed by the ith solution. The values produced in the form of a vector for the objective function are specified by Eq. (47).

$$F={\left[\begin{array}{c} F_{1}\\ F_{i}\\ F_{N} \end{array}\right]}_{N\times 1}={\left[\begin{array}{c} \left.{F(X}_{1}\right)\\ \left.{F(X}_{i}\right)\\ \left.{F(X}_{N}\right) \end{array}\right]}_{N\times 1},$$ (47)

where ***F*** represents the vector of objective function values and ***Fi*** denotes the value of the objective function for the ith solution. Each iteration involves two updates for the ZOA population members.

Phase 1: Foraging behavior

Zebra behavior models through forage seeking are employed to update the population members during the first phase (Pastor et al., 2006). Equations (48, 49) can be used in the mathematical model to update the positions of the zebras during the foraging phase.

$$x_{i,j}^{new,P1}=x_{i,j}+r\cdot \left({PZ}_{j}-I\cdot x_{i,j}\right),$$ (48)

$$X_{i}=\left\{\begin{array}{ll} X_{i}^{new,P1}, & F_{i}^{new,p1};\\ X_{i,} & \;else, \end{array}\right.$$ (49)

where $F_{i}^{new,P1}$ is the objective function value ***;*** $x_{i,j}^{new,P1}$ is ith zebra’s new status based on the first phase; *r* is a random number in the interval [0,1]. The best member is ***PZ***, the pioneer zebra; its *j*th dimension is ${\;PZ}_{j\;}$ ; ***I = round*** (***1+rand***) is provided; and rand is a random value within the interval [0,1]. Thus, ***I*** ∈ {1, 2}, and the population movement changes more noticeably if I = 2.

Phase 2: Predator defense strategies

**FIGURE 8:**
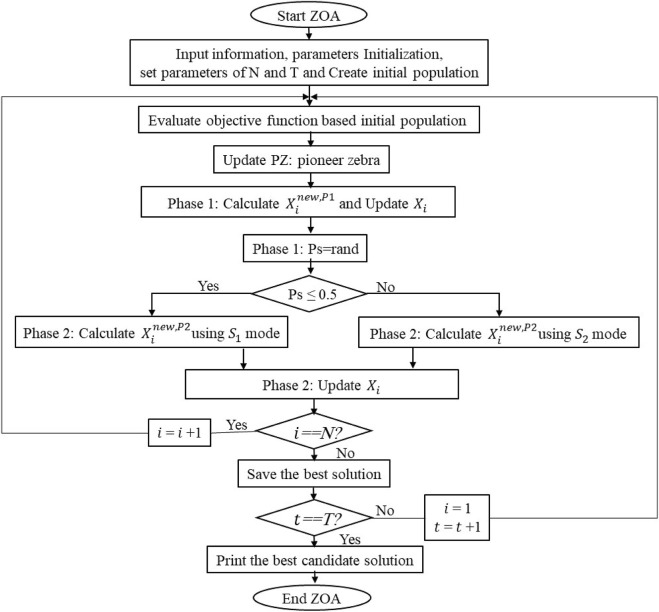
Flowchart of the ZOA.

The initial strategy for defense involves lions attacking the zebras, but the zebras move away from their current locations to escape (Pastor et al., 2006). Therefore, the mode S1 in Eq. (50) can be used to mathematically represent this strategy. In the second technique, when other predators attack one of the zebras, the other zebras in the herd move toward the attacked zebra in an attempt to confuse and intimidate the predator by forming a protective structure (Caro et al., 2014). Mode S2 in Eq. (50) is used to formally represent the zebra behaviors. When a zebra is updated, its new location is accepted if its objective function has a better value (Kennedy and Kennedy, 2013). This update scenario is represented using Eq. (51).

$$x_{i,j}^{new,P2}=\left\{\begin{array}{ll} S_{1}:x_{i,j}+R\cdot \left(2r-1\right)\cdot \left(1-\frac{t}{T}\right)\cdot x_{i,j}, & P_{s}\leq 0.5;\\ S_{2}:x_{i,j}+r\cdot \left({AZ}_{j}-I.x_{i,j}\right), & else, \end{array}\right.$$ (50)

$$X_{i}=\left\{\begin{array}{ll} X_{i}^{new,P2} & F_{i}^{new,P2}<F_{i;}\\ X_{i}, & else, \end{array}\right.$$ (51)

where *Ps* is the probability of choosing one of two randomly generated strategies in the interval [0, 1], *AZ* is the status of the attacked zebra and ${AZ}_{j}$ is its *j*th dimension value, $F_{i}^{new,P2}$ is its objective function value, and $X_{i}^{\text{new},P2}$ is the new status of the *i*th zebra based on the second phase. The iteration contour is denoted by ***t***, maximum number of iterations is given by ***T***, and constant number ***R*** is set to 0.01. The value of its *j*th dimension is $X_{i}^{new,P2}$ . The population members are updated depending on the first and second phases in each ZOA iteration. Until the time the algorithm is completely implemented, the population is updated based on Eqs. (48–51). During subsequent iterations, the best candidate solution is updated and preserved. When the ZOA is fully operational, the best potential answer is made available as the ideal response, as shown in the pseudocode and flowchart representations of the ZOA phases in Figure 8.

### 4.1 Pseudocode of the proposed ZOA

Begin ZOA.

Input: Information regarding the optimization issue.

Calculate the population size (***N***) and total number of iterations (***T***).

Evaluate the objective function based on the initial solution.

For ***t = 1: T***, update ***PZ***.

For ***i = 1: N***

Phase 1: Foraging behavior

Use Eq. (48) to determine the new ith solution.

Use Eq. (49) to update the ith solution.

Phase 2: Predator defense strategies

Ps = rand if *Ps* < 0.5.

Strategy one: Lion-fighting phase

Use mode *S1* in Eq. (50) to determine the new ith solution.

Else

Strategy two: Exploratory phase against other predators

Determine the ith zebra as a new status using mode *S2* in Eq. (50).

End if

Use Eq. (51) to update the ith solution.

Ending for *i* = 1: *N*

Save the best possible candidate solution.

Ending for *t* = 1: *T*

Display the optimal ZOA solution as the output for the given optimization problem.

Stop ZOA .

**FIGURE 9:**
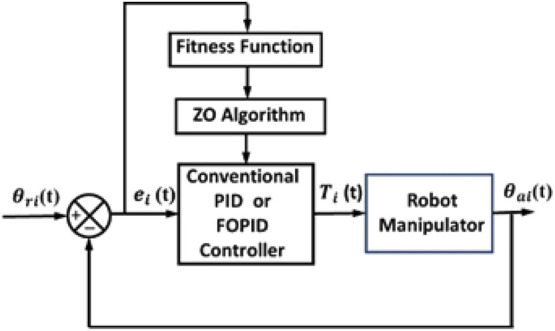
Block schematic diagram for tuning of a PID controller.

The five key components for tuning a PID controller are the fitness function, ZOA optimization method, PID controller, process, and sensor (feedback). Any controller type can be built using various optimum control parameters (Wilson et al., 2018). For the objective function (fitness function) to be minimized, some parameters must be calculated (Oleiwi, 2014). The optimization problem can be expressed using the following concepts that minimize the objective function and applied to the following constraints: ${\;K}_{p}\min<K_{p}<K_{p}\max$ , $K_{i}\min<K_{i}<K_{i}\max$ , $K_{d}\min<K_{d}<K_{d}\max$ (Baruh et al., 2002). The ZOA is used to modify the parameters of each proposed controller to minimize tracking errors between the actual and projected 2-LRRM trajectories. Figure 9 shows a block diagram of a tuned PID controller.

## 5 Simulation and results

The performances of the 2-LRRM with the proposed controllers for trajectory tracking are examined and discussed in this section. The six proposed controllers, namely W-PID, W-FOPID, RNN-PID, RNN-FOPID, NN + PID, and NN + FOPID controllers, are compared against each other to minimize the performance index when the nominal model is used. Two starting points are used for $\theta_{1}$ (Theta-1: 0.1745, 0.1745) and $\theta_{2}$ (Theta-2: −0.1745, –0.1745) to increase the learning of the controllers. The ZOA is used to find the optimal controller’s parameters that minimize the ITSE between the calculated and reference trajectories of the 2-LRRM. The settings for the ZOA are population size = 100 and maximum number of iterations = 1000. The optimal solution obtained from the last iteration is regarded as the final solution. The step size for simulation is taken as h = 0.001 s, and the simulation time is taken as 4 s. There are several ways to represent the fractional differentiation and integration components mathematically. The approximation of a fractional operator used in the design of the FOPID controller is Oustaloup’s approximation of the fifth order (N = 5) with a frequency range of [0.001, 1000] rad/s. Each link’s trajectory tracking is determined previously to allow following by the manipulator. The controller with the lowest ITSE value is considered as the best one, and the ITSE is calculated using Eq. (52).

$$ITSE=\int \left(t\times e_{1}^{2}\left(t\right)+t\times e_{2}^{2}\left(t\right)\right)dt$$ (52)

where $e_{1}\left(t\right)$ and $e_{2}\left(t\right)$ are the differences between the reference trajectories for link1 $\theta_{ri}$ and calculated trajectories for link2 $\theta_{i}$ .

One of the important advantages of a NN is its flexibility to capturing complex underlying data structures. In the design of NN controllers, this allows production of the most complex control signals with high frequencies (i.e., chattering). In fact, a chattering signal cannot be applied practically, and the optimal solution obtained is not a feasible solution. Therefore, the objective function is modified as demonstrated in Eq. (53)

Objective Function Value or Fitness Value=ITSE+ρ×Sn, (53)

where ***Sn*** is the number of times that the control signal’s slope changes signs, and ρ is a small constant number chosen as $10^{-8}$ in this work.

This modified objective function excludes any solutions that contain high chattering control signals from among the candidate solutions. The desired trajectories $\theta_{r1}$ and $\theta_{r2}$ for link1 and link2 are given in Eqs (54, 55), respectively:

$$\theta_{r1}=\left\{\begin{array}{l} {0.75\times t}^{2}-0.25\times t^{3\;},\;0<t<2\;\\ -1.5+3\times t-1.125\times t^{2}+0.125\times t^{3},2<t<4 \end{array}\right\},$$ (54)

$$\theta_{r2}=\left\{\begin{array}{l} 1.5\times t^{2}-0.5\times t^{3},\;0<t<2\\ 12-12\times t+4.5\times t^{2}-0.5\times t^{3},2<t<4 \end{array}\right\}.$$ (55)

Now that all details about the simulation and nominal model are available and known, we start applying the ZOA optimizer to adjust the gains of all the suggested controllers based on the nominal model to minimize the ITSE. Since the ZOA is a stochastic algorithm, each controller is simulated 10 times to derive the best results. Table 2 shows the ITSE values for all proposed control structures when applied to a nominal plant and executed with two initial positions.

**TABLE 2:** ITSE values of the suggested controllers for a nominal plant when using two initial positions [0.1745, 0.1745] and [−0.1745, –0.1745].

Controller	ITSE	Controller	ITSE
W-PID	9.4864 × 10^−5^	W-FOPID	8.1697 × 10^−5^
RNN-PID	9.0165 × 10^−5^	RNN-FOPID	8.6837 × 10^−5^
NN + PID	9.0259 × 10^−5^	NN + FOPID	7.9676 × 10^−5^

**FIGURE 10:**
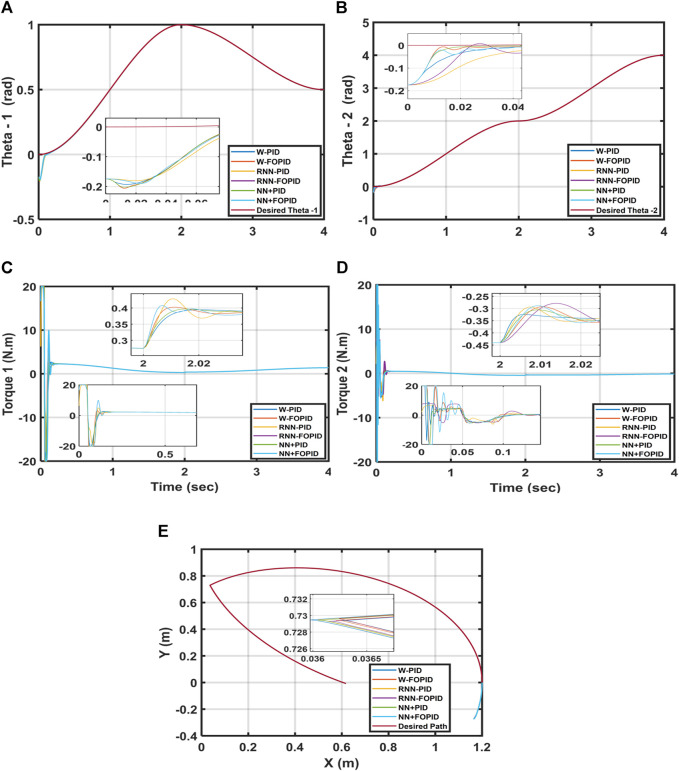
**(A)** Desired and calculated ${\;\theta }_{1}$ , **(B)** desired and calculated $\theta_{2}$ , **(C)** torque ${\;T}_{1\;}$ , **(D)** torque ${\;T}_{2\;}$ , and **(E)** end-effector trajectories of the 2-LRRM.

Overall, the findings show that in terms of the ITSE, the suggested controllers with fractional-order integral and derivative actions perform better than those with corresponding integer-order actions. This is attributed to the fact that the tuning parameters of the controller are increased by the FOPID, which in turn increases the number of DOFs, controller capabilities, and robustness. Despite the results being very close to each other, as seen from Table 2, the findings indicate that the hybrid NN + FOPID structure has the highest ITSE of 7.9676 × 10^−5^ while the W-PID controller has the lowest ITSE of 9.4864 × 10^−5^. Figure 10 shows the trajectory tracking performances of $\theta_{1}$ **,** $\theta_{2}$ (the paths followed by the 2-LRRM’s end effectors), and the suggested controllers’ torques **𝑇**_**1**_ and 𝑻_**2**_.

Based on the results, it is concluded that the NN + FOPID controller performs better than all the other suggested controllers and that it is the best controller among them.

## 6 Robustness tests

This section presents the results of the proposed controllers that are subjected to robustness tests. To show the capabilities of each controller, the following experiments are implemented in MATLAB without adjusting the gains or retuning the gains of the proposed controllers.

### 6.1 Initial condition changes

**FIGURE 11:**
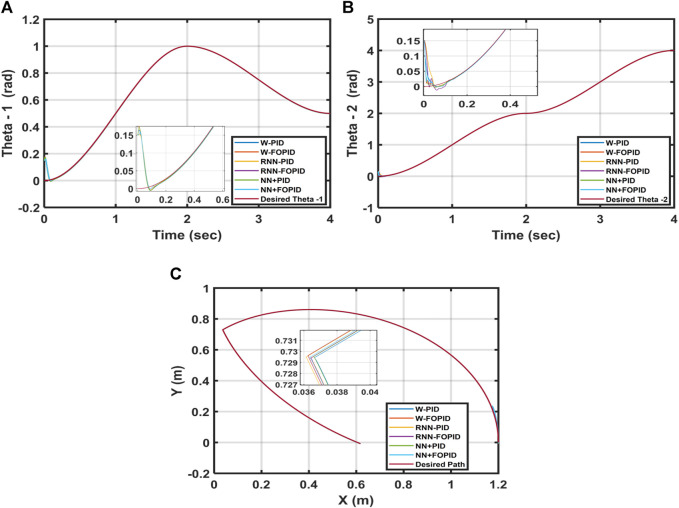
**(A)**Desired and calculated ${\;\theta }_{1}$ , **(B)**desired and calculated ${\;\theta }_{2}$ , and **(C)**2-LRRM end effector trajectories when the starting point is [0.15, 0.15].

In this test, another set of initial conditions [0.15, 0.15] was considered for [ $\theta_{1}$ , $\theta_{2}$ ] to evaluate each controller’s robustness and test its ability to follow the required trajectory of the 2-LRRM. Table 3 presents the ITSE values for each of the suggested controllers. Figure 11 depicts the trajectory tracking of *θ_1_* and *θ_2_* and the end-effector of the 2-LRRM by changing the initial location for all suggested controllers.

**TABLE 3:** ITSE values of the proposed controllers for an initial position of [0.15, 0.15].

Controller	ITSE	Controller	ITSE
W-PID	3.6563 × 10^−5^	W-FOPID	2.5491 × 10^−5^
RNN-PID	2.5536 × 10^−5^	RNN-FOPID	2.6496 × 10^−5^
NN + PID	3.1708 × 10^−5^	NN + FOPID	2.3871 × 10^−5^

Despite altering the starting position, the NN + FOPID controller performs better than the other suggested controllers, where the ITSE = 2.3871 × 10^−5^ of the NN + FOPID controller is the optimal among them. Furthermore, in the trajectory response, NN + FOPID has the least amount of overshoot and fastest settling time for ${\;\theta }_{1}$ as well as a good overshoot and good settling time for ${\;\theta }_{2}$ . The W-PID has the worst ITSE of 3.6563 × 10^−5^, and its responses are poor owing to its large overshoot and lengthy settling times for the ${\;\theta }_{1}$ and $\theta_{2}$ responses. Furthermore, the trajectory of the 2-LRRM end effector for the NN + FOPID controller still follows the most similar path as the desired trajectory.

### 6.2 Parameter variations

**FIGURE 12:**
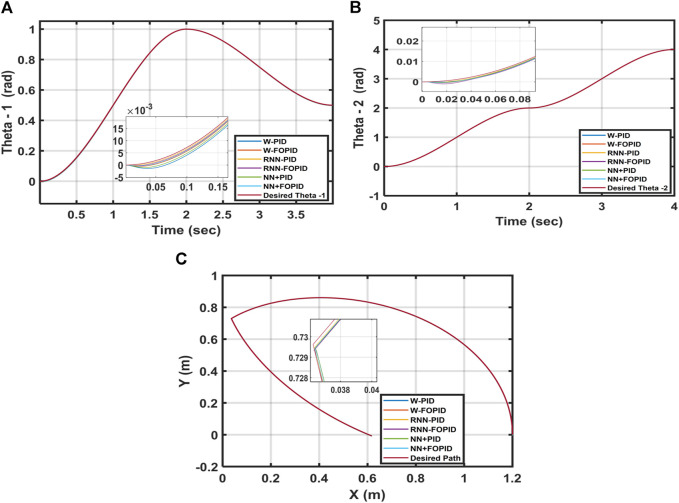
Desired and calculated trajectories for **(A)** ${\;\theta }_{1}$ , **(B)** ${\;\theta }_{2}$ , and **(C)** 5% added masses to both links for a starting position of [0, 0].

In this test, the parameter variations of the 2-LRRM model are investigated for the suggested control structures by incrementing the mass of each link by 5%. Table 4 shows the ITSE value of each controller. The RNN-PID controller has the best performance index of ITSE = 0.1418 × 10^−5^, and the tracked trajectories for $\theta_{1}$ and ${\;\theta }_{2}$ are closest to the required trajectories of ${\;\theta }_{r1}$ and ${\;\theta }_{r2}$ than the other suggested control structures. Consequently, when using the RNN-PID controller, the trajectory followed by the 2-LRRM end effector with parameter variations is very close to the desired trajectory. The second-best controller is the NN + FOPID, with a performance index of ITSE = 0.3139 × 10^−5^ and good trajectory tracking for $\theta_{1}$ and ${\;\theta }_{2}$ ; the worst controller is the W-PID with ITSE = 1.0759 × 10^−5^, and its trajectory tracking has large overshoots and lengthy settling times. Figure 12 shows the trajectories tracked for $\theta_{1}$ and $\theta_{2}$ as well as the 2-LRRM end effector when the link masses are changed for each controller.

**TABLE 4:** ITSE values of the proposed controllers when adding 5% to the masses of both links and setting the starting position to [0.0, 0.0].

Controller	ITSE	Controller	ITSE
W-PID	1.0759 × 10^−5^	W-FOPID	0.4012 × 10^−5^
RNN-PID	0.1418 × 10^−5^	RNN-FOPID	0.3324 × 10^−5^
NN + PID	0.7397 × 10^−5^	NN + FOPID	0.3139 × 10^−5^

### 6.3 Disturbance addition

**FIGURE 13:**
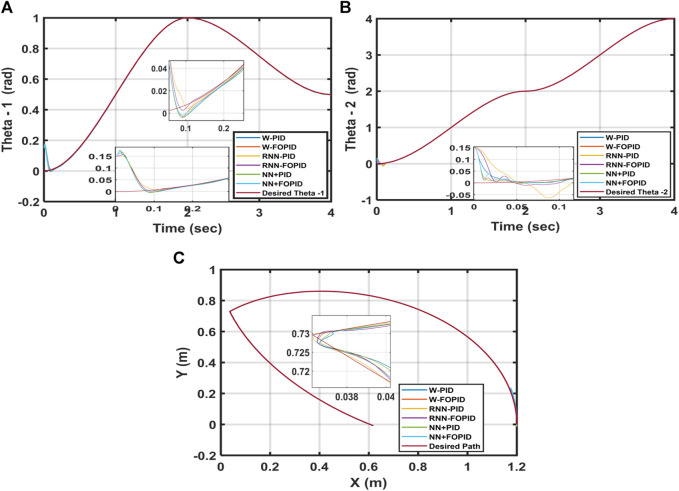
Desired and calculated trajectories for **(A)** ${\;\theta }_{1}$ , **(B)** $\theta_{2}$ , and **(C)** end effector for an added disturbance of [sin(50t), sin(50t)] and starting point of [0.15, 0.15].

Another test was conducted to determine the robustness of the proposed control structures by increasing the disturbance terms [sin (50t), sin (50t)] in the control actions [ $T_{1}$ , $T_{2}$ ] and setting the initial positions as [0.15,0.15] for [ ${\;\theta }_{1}$ , ${\;\theta }_{2}$ ]. Table 5 shows the obtained ITSE values of the proposed controllers. The trajectories tracked for ${\;\theta }_{1}$ and ${\;\theta }_{2}$ as well as the end effector of the 2-LRRM by increasing the disturbance term by sin(50t) N∙m in both links are demonstrated in Figure 13. From the results, it is concluded that the NN + FOPID controller performs the best in terms of disturbance rejection when compared to the other controllers. The NN + FOPID controller is also the best in terms of the ITSE and has the smallest overshoot during trajectory tracking.

**TABLE 5:** ITSE values of the suggested controllers with added disturbance of sin(50t) to both links and a starting position of [0.15, 0.15].

Controller	ITSE	Controller	ITSE
W-PID	4.7128 × 10^−5^	W_FOPID	3.7435 × 10^−5^
RNN-PID	4.2479 × 10^−5^	RNN-FOPID	4.9497 × 10^−5^
NN + PID	4.0179 × 10^−5^	NN + FOPID	3.4328 × 10^−5^

### 6.4 All tests conducted simultaneously

**FIGURE 14:**
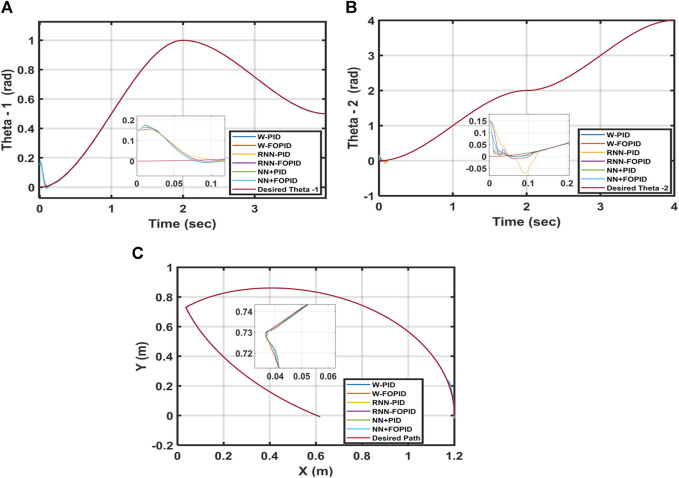
Desired and calculated trajectories for **(A)** ${\;\theta }_{1}$ , **(B)** $\theta_{2}$ , and **(C)** end effector when using a starting point of [0.15, 0.15], adding a disturbance of sin(50t) to the torques of both links, and adding 5% to the masses of both links.

This combined test is crucial when evaluating robustness since it determines which of the proposed controllers can be used as the best controller. All suggested controllers are subjected to the combined impacts of added disturbance of sin(50t) to the control signals [ ${\;T}_{1\;}$ , ${\;T}_{2}$ ], increasing the masses of the two links by 5%, as well as altering the starting points to [0.15, 0.15]. Table 6 displays the ITSE value of each controller based on the results attained. Among all the suggested controllers, the lowest ITSE is observed for the NN + FOPID controller. Figure 14 shows the trajectories tracked for $\theta_{1}$ and ${\;\theta }_{2}$ as well as the end effector of the 2-LRRM for disturbance additions, parameter variations, and initial position changes for all proposed controllers. The NN + FOPID controller still shows optimal results and performs better than all the other proposed controllers because the trajectory followed by the 2-LRRM end effector and tracked the trajectories of $\theta_{1}$ and ${\;\theta }_{2}$ for the NN + FOPID controller are all close to the required trajectories.

**TABLE 6:** ITSE between the desired and calculated paths when using a starting point of [0.15, 0.15] with added disturbances of [sin(50t), sin(50t)] and added masses of 5% to both links.

Controller	ITSE	Controller	ITSE
W-PID	4.9523 × 10^−5^	W-FOPID	3.7390 × 10^−5^
RNN-PID	4.8589 × 10^−5^	RNN-FOPID	5.1111 × 10^−5^
NN + PID	4.2179 × 10^−5^	NN + FOPID	3.5742 × 10^−5^

## 7 Conclusion

In this study, six PID- and FOPID-based control structures are proposed for a 2-LRRM for trajectory tracking; these are named W-PID, W-FOPID, RNN-PID, RNN-FOPID, NN + PID, and NN + FOPID controllers. To optimize the controller parameters, the ZOA was used offline to minimize the performance index ITSE. The MATLAB simulation results show that all proposed controllers have the ability to quickly reduce the errors between the real and desired paths before tracking the required path. By altering the initial state values, adding disturbances to the control signals, and increasing the masses of the two links, the robustness of each suggested controller was examined. According to the results of the nominal system tuning test and all robustness tests, the NN + FOPID has the best control structure among the suggested controllers. The combination of NN with fractional-order integration and differentiation affords high performance and efficient responses, which are reflected in the results. In addition, the modified objective or fitness function allowed ZOA-based tuning of the controller parameters to determine stable responses with fewer fluctuations in the control signals. The trajectory tracking responses for Theta-1 and Theta-2 have the smallest overshoots, shortest settling times, and lowest ITSE values. Moreover, the NN + FOPID controller outperformed all the other controllers in the robustness tests. This work also highlights the capacity of the ZOA for fine-tuning the controller parameters. Finally, as a future work, this study can be extended using other optimization techniques instead of the ZOA, such as the firebug swarm optimization algorithm, chimp optimization algorithm, mayfly optimization algorithm, and gazelle optimization algorithm to adjust the controller gains. In addition, a real robot manipulator equipped with all required hardware may be employed to practically implement and verify the suggested controllers.

## Data Availability

The original contributions presented in the study are included in the article/supplementary material; further inquiries can be directed to the corresponding author.
